# Ferulic acid attenuates microglia-mediated neuroinflammation in retinal degeneration

**DOI:** 10.1186/s12886-020-01765-7

**Published:** 2021-01-06

**Authors:** Xiaowei Sun, Peng Sun, Limei Liu, Pengfei Jiang, Yuanbin Li

**Affiliations:** Department of Ophthalmology, The Affiliated Yantai Yuhuangding Hospital of Qingdao University, Yantai, 264000 People’s Republic of China

**Keywords:** Microglia, Retinal degeneration, Inflammation, Ferulic acid, IRF8

## Abstract

**Background:**

Retinal degeneration is often accompanied by microglia-mediated neuroinflammation. Ferulic acid (FA), an active ingredient of traditional Chinese medicines (TCMs), has been reported to have anti-inflammatory effects. This study explores the impact of FA on microglia-mediated neuroinflammation and associated retinal degeneration in rd10 mice.

**Methods:**

Rd10 mice received different concentrations of FA every day from postnatal day (P)4 to P24. On P25, the visual function of the mice was evaluated by electroretinogram, and retinae were collected for further investigation. Microglial activation and the expression of relevant cytokines in the retina were evaluated by qPCR, western blotting and immunofluorescence staining. Retinal structure was assessed by haematoxylin and eosin (HE) staining.

**Results:**

Supplementation with 50 mg/kg FA provided optimal protection against retinal degeneration, with treated mice exhibiting more photoreceptor nuclei as well as greater wave amplitude amplification on electroretinogram than untreated mice. FA suppressed microglial activation both in vivo and in vitro, and inhibited the expression of pro-inflammatory factors Tnfα, IL1β, and Ccl2 in the retinae of rd10 mice. Furthermore, FA suppressed the activation of STAT1 and subsequently inhibited IRF8 expression, potentially highlighting a role for these pathways in FA-mediated immunomodulatory activity.

**Conclusions:**

Attenuation of neuroinflammation by FA may be beneficial for retarding retinal degeneration.

**Supplementary Information:**

The online version contains supplementary material available at 10.1186/s12886-020-01765-7.

## Background

Progressive neuronal loss is responsible for symptom onset in retinal degeneration diseases, the most common of which is retinitis pigmentosa (RP), which is the leading cause of inherited retinal degeneration-associated blindness and affects approximately 1.5 million people worldwide [1]. With the advent of next-generation sequencing and recent advances in gene therapy, an increasing number of gene mutations responsible for RP have been identified [2]. Gene therapy for RP is limited by the heterogeneous genetic basis of this disease, highlighting the importance of developing therapies that act independently of mutation status [3]. Drugs targeting the broad pathological processes that are common to different kinds of RP caused by various mutations may be beneficial for this complex disorder [4].

Neuroinflammation is now widely recognized to participate in many chronic neurodegenerative diseases, including multiple sclerosis, Alzheimer’s disease, and Parkinson’s disease, and attenuation of neuroinflammation is an effective therapeutic approach for these diseases [5]. There is increasing evidence that microglia, the resident immune cells in the retina, induce immune responses and create a chronic inflammatory environment in the retinae of RP patients [6]. The anterior vitreous cavities of RP patients were found to contain many inflammatory cells, and the levels of various pro-inflammatory chemokines and cytokines are upregulated in the aqueous humour and vitreous fluid in individuals with this disease [7], suggesting a role for inflammation in the process of retinal degeneration. Research findings provide strong support for the concept that microglia-mediated neuroinflammation contributes to the overall apoptosis of photoreceptors in the retinae of rd10 mice [8].

Ferulic acid (FA), a phenolic compound present in the plant wall, is a major active ingredient of some traditional Chinese medicines (TCMs), such as *Ferula asafetida*, *Angelica*, and *Ligusticum wallichii*, which are TCM prescriptions used to improve microcirculation in ischaemic diseases [9, 10]. Increasing research has demonstrated that FA suppress detrimental immunoreactions under various conditions. FA is easily obtainable and has good application prospects for the treatment of Alzheimer’s disease (AD) based on its potent immunomodulatory properties [11, 12]. In an ovalbumin-induced model of respiratory allergies, FA administration was shown to dampen Th2-mediated immunity [13]. These research results indicate that FA may be a novel immunosuppressive agent. As microglia-mediated neuroinflammation is believed to be one of the key processes responsible for neurodegeneration in RP and related diseases, immunosuppression targeting microglia is a promising strategy for the treatment of these diseases. We therefore speculate that FA may be capable of slowing or arresting the retinal degeneration process owing to its ability to suppress microglia-mediated neuroinflammation.

In our study, the effect of FA immunomodulation on the pathological process of retinal degeneration in the rd10 mouse model of RP was evaluated. The results showed that FA suppressed the transformation of microglia into a reactive phenotype and rescued retinal degeneration in rd10 mice. The underlying mechanism may have been FA-induced suppression of the expression of interferon regulatory factor 8 (IRF8), a key factor that promotes microglial activation, and thus a reduction in the production of inflammatory cytokines.

## Methods

### Rd10 mice and FA treatment

Rd10 mice (The Jackson Laboratory) were housed in a specific pathogen-free facility in the Animal Laboratories of Yantai Yuhuangding Hospital. All animal studies adhered to the ARVO Statement for Use of Animals in Ophthalmic and Vision Research. It has been reported that in rd10 mice, retinal abnormalities begin on postnatal day (P)7 [14, 15] and that photoreceptor death peaks around P25 and is nearly complete by P35 [16, 17]. For treatment, rd10 animals were intragastrically administered 25 mg/kg, 50 mg/kg or 100 mg/kg FA (1,270,311, Sigma) every day from P4 to P24. Before intragastric administration, the mice were anaesthetized with isoflurane in animal anaesthesia ventilator system (Matrx, America); the mice experienced no pain and awoke quickly. The animals were sacrificed via sodium pentobarbital injection (200 mg/kg; intraperitoneal) on P25, and their eyeballs were enucleated for further investigation.

### Electroretinogram (ERG) recordings

Before ERG recordings, the mice were adapted to the dark overnight. The mice were anaesthetized by intraperitoneal pentobarbital sodium (50 mg/kg) prior to pupil dilation using 1% tropicamide. ERGs were recorded with a Ganzfeld stimulator (Roland Consult, Germany) that generated and controlled light stimuli. Scotopic ERGs were recorded following a 1.3-ms single flash with an intensity of-1.52, − 0.52, 0.48 or 1.0 log cd s/m^2^. A total of 5 responses per intensity were averaged for each flash stimulus. Intraperitoneal sodium pentobarbital (200 mg/kg) was then used to euthanize the mice. The amplitudes of the major ERG components (a- and b-waves) were measured (RETI System software) using automated and manual methods.

### Hematoxylin and eosin (HE) staining

Eyeballs were fixed in formalin overnight prior to being embedded in and sectioned into 3-μm slices. Before staining, xylene was used for deparaffinization, and the sections were rehydrated in an ethanol gradient prior to being washed in PBS. Then, the sections were stained with HE. A microscope (Leica DM4000, Germany) was used to analyse retinal histology and count nuclei in the outer nuclear layer (ONL).

### Cell culture and FA treatment

BV2 murine microglial cells (purchased from cell bank, Kunming Institute of Zoology) were cultured as previously described [18]. In brief, the cells were maintained in DMEM (high-glucose) containing 10% FBS and penicillin/streptomycin. The microglia were activated by lipopolysaccharide (LPS, 50 ng/ml, L6529, Sigma) for 1 h and then treated with FA (0.05 mg/mL, 0.1 mg/mL, 0.2 mg/mL, 0.5 mg/ml, 1 mg/ml, 2 mg/ml or 5 mg/ml, 6529, Sigma). After 24 h, the cells were collected for downstream analyses.

### Immunofluorescence staining

For retinal wholemounts, eyes were immersed in 4% paraformaldehyde (PFA) fixative for 30 min, and the retinal cups were separated carefully from the eyeballs. Both retinal wholemounts and cell slides were stained using primary and secondary antibodies, washed extensively and flat-mounted. The following primary antibodies were used: anti-iba1 (019–19,471, Wako Chemicals) and anti-iNOS (sc-7271, Santa Cruz). The following secondary antibodies were used: Alexa Fluor 488-conjugated donkey anti-rabbit IgG H&L and Alexa Fluor 555-conjugated donkey anti-goat IgG H&L. The retinal wholemounts and cell slides were visualized via confocal microscopy (Carl Zeiss LSM710, Germany).

### RNA sequence

RNA was isolated from retinae using TRIzol Reagent (Invitrogen), and a Bioanalyzer 2100(Agilent) was used to gauge the quality of the resultant nucleic acid. RNA preparation, library construction, and sequencing were conducted using a BGISEQ-500 instrument at the Beijing Genomics Institute (BGI, Shenzhen, China).

### RT-PCR

Total RNA was isolated from the retina of rd10 using the RNAiso Plus kit (TAKARA Bio Inc., Japan), and the Reverse Transcriptase Superscript II Kit (TAKARA Bio Inc., Japan) was then used to prepare cDNA following the instructions. Rea-time PCR was performed in a 20-μL reaction system, containing 10 μL of 2 × SYBR Premix Ex Taq, 2 μL of cDNA, and 10 μmol/L primer pairs. The thermocycler settings were 95 °C for 30 s and 40 cycles of 95 °C for 5 s and 60 °C for 34 s.

### Western blot analysis

RIPA buffer (Biocolors, Shanghai, China) containing dissolved protease and phosphatase inhibitor mini tablets (Thermo Fisher Scientific, MA, USA) was used to lyse homogenized retinal tissue. The samples were then centrifuged for 10 min at 10,000 rpm, after which a BCA assay was used for protein quantification. Equivalent amounts of protein were utilized for western blotting. The blots were incubated overnight in primary antibodies, including anti-STAT1, anti-pSTAT1(14994S, 7649S, CST), anti-IRF8(sc-365,042, SANTA) and β-actin (ab28696, Abcam, Cambridge, MA). After being washed with PBST, the membranes were probed with HRP-linked secondary antibodies (1:2000) for 1 h at room temperature.

### Statistics

Each experiment, including the immunostaining, qPCR and western blotting experiments, was replicated 3 times. All quantitative data was analysed using 2-tailed Student’s t test or one-way ANOVA by SPSS 21.0. The data are the means ± standard errors of the mean (SEMs). *P* < 0.05 indicated significance.

## Results

### FA ameliorated retinal degeneration in rd10 mice

**Fig. 1:**
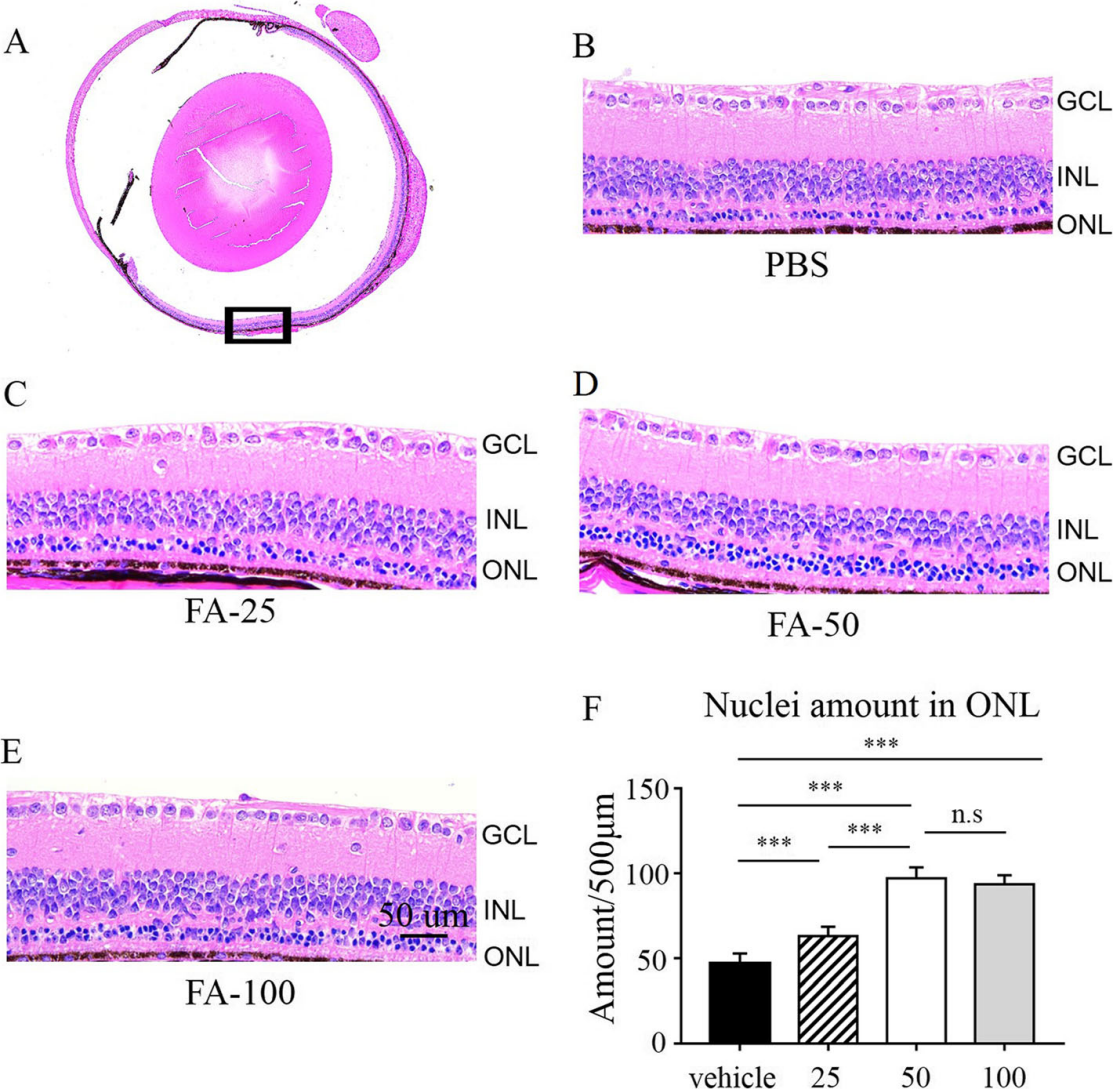
Histologic analysis of retinal thickness after FA treatment. **a** Full view of the retinal mid-peripheral area following HE staining. GCL: ganglion cell layer; INL: inner nuclear layer; ONL: outer nuclear layer. **b-e** FA-treated rd10 mice exhibited an increase in the thickness of the ONL (scale bar, 50 μm). **f** Statistical analysis of the number of nuclei in the ONL revealed that FA supplementation delayed retinal degeneration in rd10 mice (***p < 0.001, *n* = 6 eyes)

FA has shown some efficacy in brain disorders and neurodegenerative diseases. As nervous tissue, the retina is a continuation of brain; thus, we evaluated the effect of FA on retinal degeneration in rd10 mice. Because photoreceptor death peaks around P25 [16], we intragastrically administered different doses of FA (25 mg/kg, 50 mg/kg, and 100 mg/kg) to rd10 mice every day from P4 to P24. HE staining of retinal sections was performed on P25 to evaluate retinal structure. As shown in Fig. 1b, after treatment with PBS, there was only one row of cells in the ONL in rd10 mice on P25. Notably, 25 mg/kg/d, 50 mg/kg/d and 100 mg/kg/d FA exerted significant protective effects in rd10 mice, as rd10 mice treated with 25 mg/kg/d, 50 mg/kg/d and 100 mg/kg/d FA exhibited two, four and three rows of cells in the ONL, respectively (Fig. 1c, d and e). We also calculated the number of nuclei in the ONL, as shown in Fig. 1f. The numbers of nuclei in the ONL were 63.32 ± 3.34, 95.20 ± 3.53, 92.46 ± 3.74 per 500 μm in the 25 mg/kg, 50 mg/kg, and 100 mg/kg FA-treated groups, respectively, and 47.23 ± 3.34 in the PBS-treated group. Together, these results show that FA can prevent retinal degeneration in rd10 mice and that 50 mg/kg/d FA may be the optimal dose for further investigation.

### FA supplementation improved retinal function in Rd10 mice

**Fig. 2:**
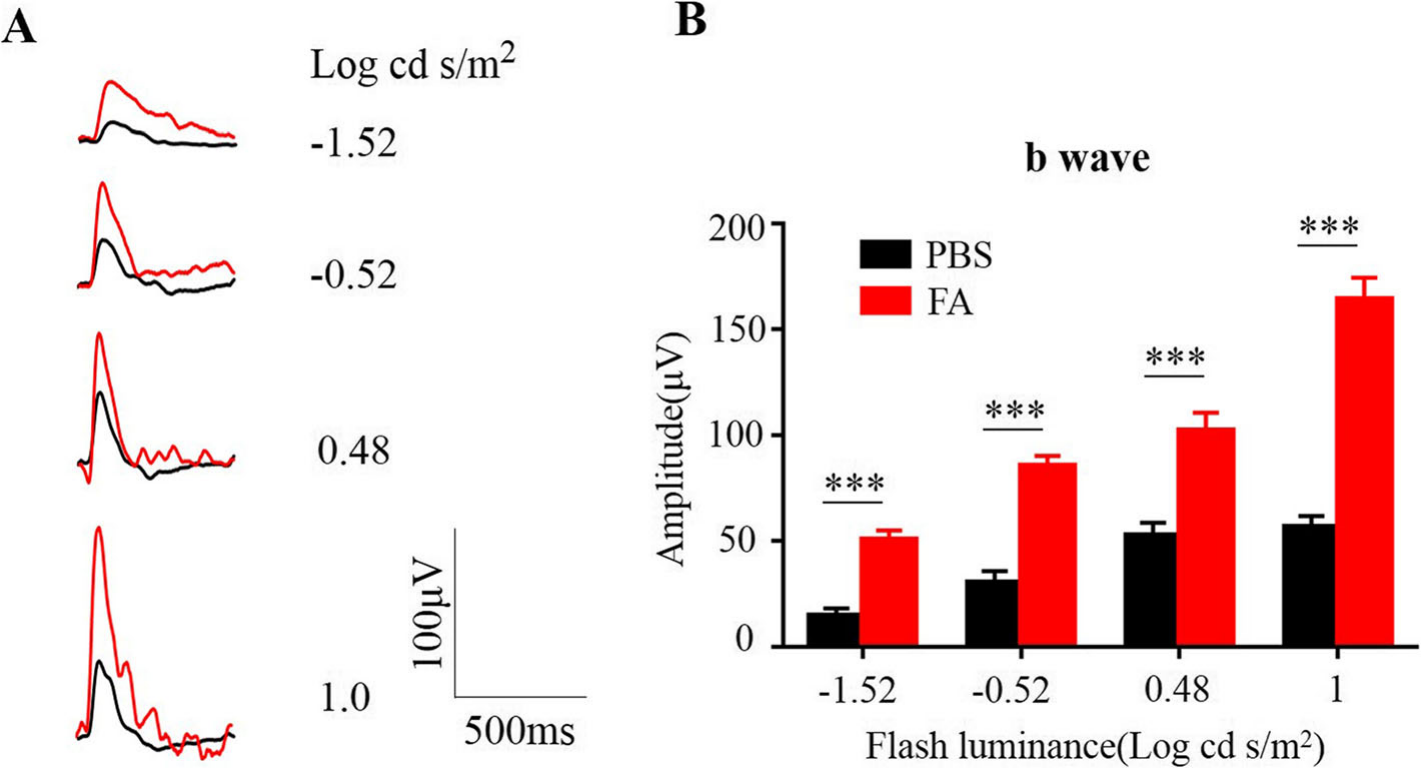
FA preserved retinal function in rd10 mice. **a** On P25, single-flash ERG recordings at light intensities of − 1.52, − 0.52, 0.48 and 1.0 log cd s/m^2^ were used to evaluate retinal function in mice. Compared with PBS-treated rd10 mice, FA-treated mice showed significant amplification of waves under multiple testing conditions. **b** Statistical analysis of b-waves in PBS- and FA-treated rd10 mice (***p < 0.001, *n* = 9 mice)

Next, we assessed the retinal function of rd10 mice by ERG on P25. The average b-wave amplitude of each group of rd10 mice was analysed at light intensities of − 1.52, − 0.52, 0.48 and 1.0 log cd s/m^2^ (Fig. 2a). As shown in Fig. 2b, under a variety of scotopic testing conditions, b-wave amplitudes were 50.34 ± 2.53 μV, 85.65 ± 4.54 μV, and 102.42 ± 3.28 μV and 164.44 ± 3.17 μV in the FA-treated group and 14.52 ± 1.43 μV, 30.26 ± 2.3 μV, 52.45 ± 1.08 μV, and 56.47 ± 3.27 μV in PBS-treated group, confirming that FA can protect retinal function in rd10 mice.

### FA suppressed microglial activation and retinal inflammation in Rd10 mice

**Fig. 3:**
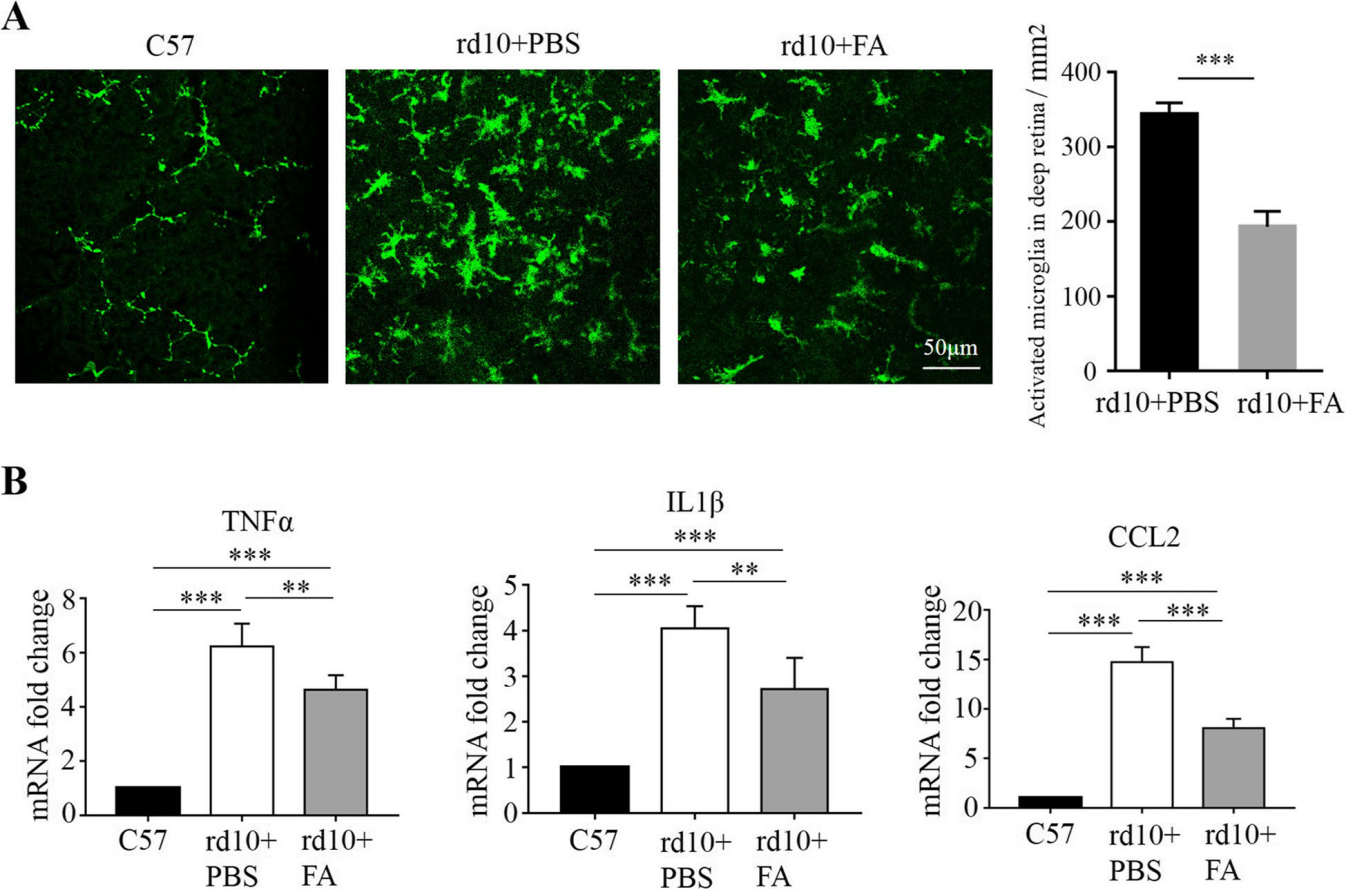
FA suppressed microglial activation and inflammation in the context of retinal degeneration. **a** In retinal whole mounts, the number of iba1^+^ activated microglia was significantly lower in the FA-treated group than in the PBS-treated group (***p < 0.001, *n* = 6 eyes; scale bar, 50 μm). **b** The mRNA levels of TNFα, IL1β, and CCl2 were suppressed after FA treatment (**p < 0.01, ***p < 0.001, *n* = 6 eyes)

Our previous study showed that more microglia are observed in the retinae of rd1 mice than in those of C57 mice and that microglia are specifically activated to a pro-inflammatory phenotype during the rapid rod degenerative phase from P14 to P28, suggesting the involvement of microglia in retinal neuroinflammation and degeneration [19]. As expected, in this study, we noticed that Iba-1^+^ microglia were activated in the retinae of rd10 mice on P25; these Iba-1^+^ microglia exhibited an amoeboid-like morphology, a swollen body and stubby branches, while ramified-resting microglia in C57 mice resembled an octopus, with a small body and long branches (Fig. 3a). After FA treatment, the number of IBA1^+^ positive microglia was significantly decreased in the retina of rd10 mice on P25 (Fig. 3a). As expected, FA suppressed the mRNA expression of chemokines and inflammatory cytokines, such as Tnfα, IL1β, and Ccl2, by inhibiting microglial activation (Fig. 3b). These results suggest that FA can alleviate the microglial inflammatory response during retinal degeneration.

### FA suppressed microglial activation in cultured BV2 cells under LPS insult

**Fig. 4:**
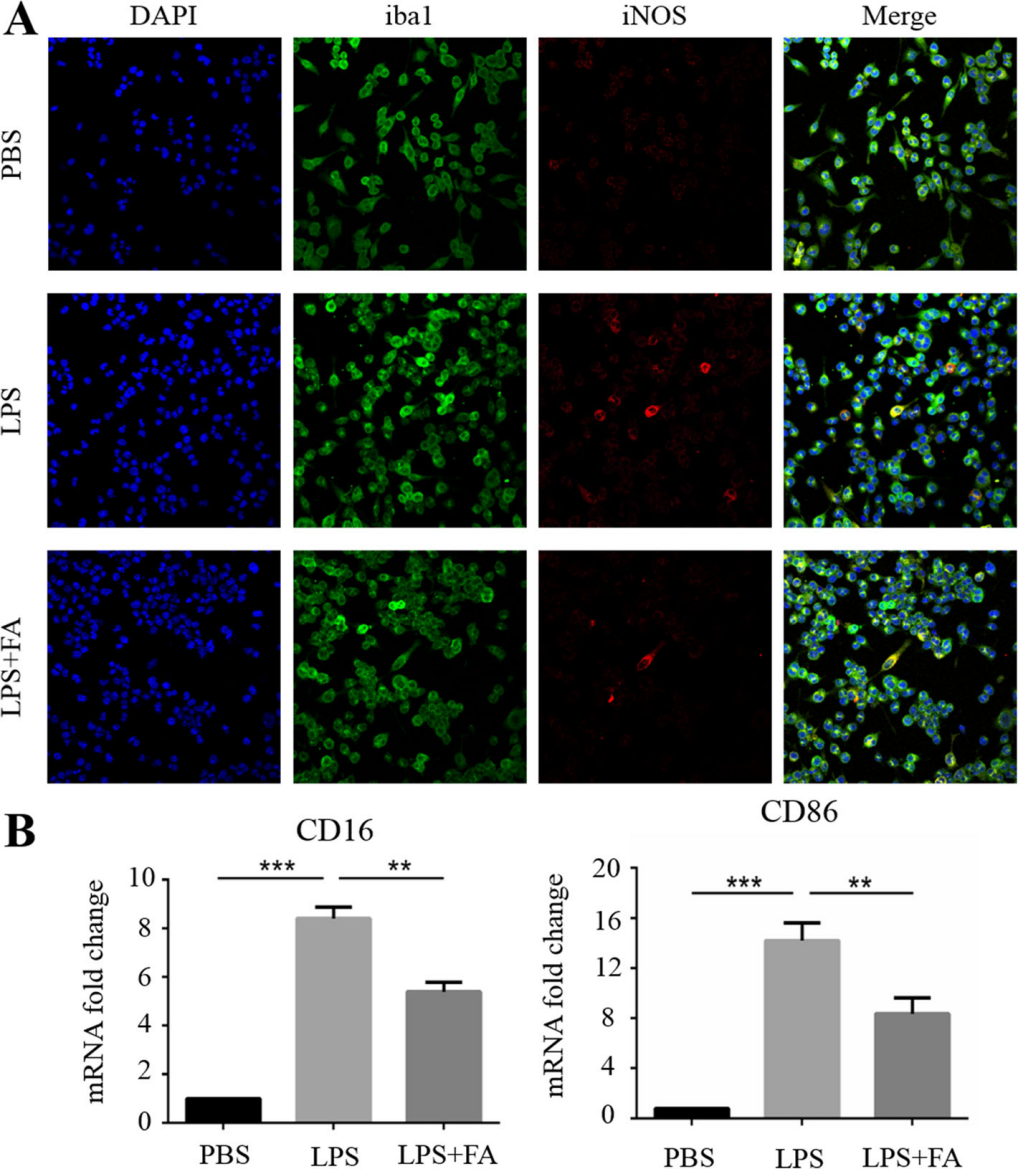
FA suppressed LPS-induced microglial activation in vitro. **a** Immuno-fluorescence staining showed numerous iNOS^+^ microglia among cultured BV2 cells under LPS stimulation but no microglial activation in the PBS-treated control cells. After FA treatment, the number of iNOS^+^ activated microglia was obviously reduced (scare bar, 40 μm). **b** FA suppressed the mRNA expression of CD16 and CD86

To explore the effect of FA on microglial activation in vitro, BV2 cells were stimulated with LPS, which is a classical agonist that triggers microglial activation. Inducible nitric oxide synthase (iNOS) is a marker of activated microglia that continuously produces NO, which is an important pro-inflammatory cytotoxic agent [20]. CD16 and CD86 are surface markers of proinflammatory microglia. iNOS mRNA expression in BV2 cells was detected to confirm the optimal dose for further investigation. As shown in Supplementary Fig. 1, 0.5 mg/mL was the minimal dose that achieved a therapeutic effect. Immunofluorescence revealed many more iNOS+ BV2 cells in the LPS treatment group than in the control group and the mRNA expression of CD16 and CD86 was obviously up-regulated, indicating that the BV2 cells adopted a reactive phenotype. After FA treatment, the number of iNOS+ BV2 cells was significantly decreased, the mRNA expression of CD16 and CD86 was suppressed (Fig. 4a, b) and NO synthesis was significantly inhibited (Supplementary Table 1). These data suggest that FA can suppress microglial activation in vitro.

### FA modulated IRF8 activation and phosphorylation of STAT1 in microglia in Rd10 mice

**Fig. 5:**
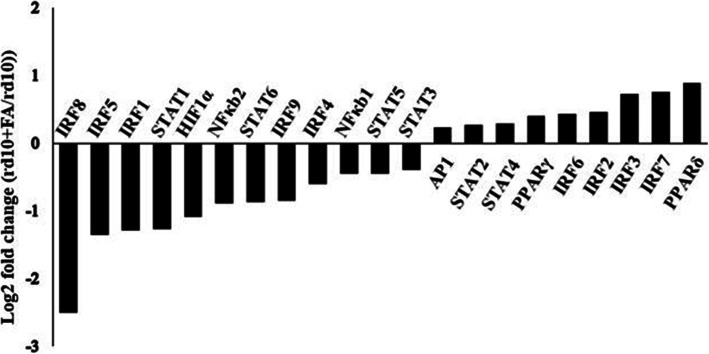
RNA-seq analysis of the retinae of rd10 mice. After FA administration, many transcription factors were differentially expressed in the retinae of rd10 mice. mRNA expression fold change = log2(rd10 + FA/rd10) (*n* = 3)

**Fig. 6:**
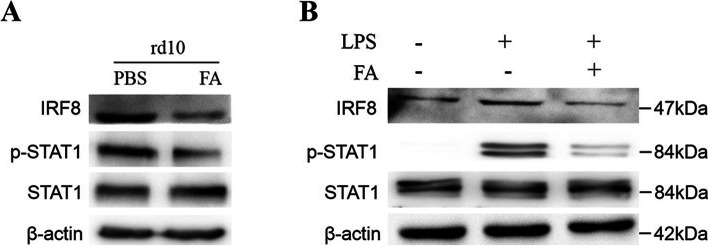
FA modulated STAT1 phosphorylation and IRF8 activation in vivo and vitro. The retina of FA-treated rd10 mice exhibited lower STAT1 signalling and IRF8 expression than PBS-treated retinae, as evaluated by western blotting. In vitro, LPS upregulated the expression of IRF8 and STAT1 phosphorylation in BV2 cells, while FA treatment reduced both STAT1 activation and IRF8 expression levels in these cells

RNA-seq analysis of the retina of rd10 mice was conducted to explore the underlying mechanism by which FA suppresses microglia-mediated inflammation. Members of the IRF family and STAT family, PPAR, AP1, NFκb and HIF1α are accepted transcription factors that may regulate microglial polarization and IRF8 mRNA expression was most obviously decreased in the retinae of rd10 mice after FA treatment in this study (Fig. 5). We next evaluated IRF8 and STAT1 signalling via western blotting and found that IRF8 expression at the protein level was significantly suppressed in the retinae of FA-treated rd10 mice. STAT1 phosphorylation was also reduced following FA treatment, leading to reduced IRF8 expression (Fig. 6a, Supplementary Figure 2). Furthermore, FA administration inhibited IRF8 expression and the phosphorylation of STAT1 in LPS-stimulation BV2 cells (Fig. 6b, Supplementary Figure 2). FA may therefore act by regulating STAT1 activation and IRF8 expression.

## Discussion

The progression of RP is largely driven by neuroinflammatory processes. Herein, we demonstrated the efficacy of FA, a Chinese herbal monomer with immunomodulatory potential. Specifically, we demonstrated that FA was able to suppress neuroinflammation and thereby slow the degenerative progression of RP in rd10 mice. Together, these findings highlight the potential value of FA or similar immunomodulatory treatments in slowing retinal degeneration in RP patients.

FA has been recognized as an important chemical with several biological activities, including direct and indirect anti-inflammatory, antioxidant, antiviral, antiallergic, antimicrobial, antithrombotic, anticarcinogenic, and hepatoprotective actions [21]. Increasing attention has been paid to the ability of FA to suppress inflammation by regulating the immune response [22–24]. FA can modulate immune activity in many cell types. For example, Cho et al. found that sustained FA treatment of mice can suppress Aβ-induced astrocyte activation, thereby preventing the production of associated inflammatory cytokines and free radicals that can drive AD-associated inflammation [25]. FA treatment attenuates dextran sulfate sodium-induced colitis in model mice and induces Treg differentiation [26]. FA can also suppress LPS-mediated IL-1β and IL-6 secretion from macrophages [27]. In this study, the results indicate that FA is able to suppress microglial activation and reduce the expression of IL-1β, IL-6 and CCL2 in rd10 mice.

Microglia are the only immune cells that normally reside in the retina in significant numbers. In the retinae of humans with RP, rod apoptotic death is associated with the migration of these microglia from the inner to the outer retina. In the rd10 mouse model of RP, infiltration of activated microglia into the subretinal space can be detected by P16 even though apoptotic death of photoreceptors is only evident at P19, indicating that microglia themselves are capable of driving the apoptosis of these cells [8]. Microglial activation contributes to inflammation through the secretion of inflammatory cytokines and chemokines that accelerate photoreceptor apoptosis, including TNF-α, IL-1β, IL-6, and CCL2 [28]. Furthermore, these cells can also phagocytose both dead apoptotic and live stressed photoreceptor cells, aggravating retinal degeneration [29–31].

Activation-induced gene expression in microglia is tightly regulated by many transcription factors [32]. IRF family proteins are thought to be essential regulators of immune cell activation and responsiveness [33]. IRF8 is almost exclusively expressed in myeloid and lymphoid cells of the immune system, with retinal IRF8 is found only in microglia [34], which share features with cells of the myeloid lineage. Changes in retinal IRF8 levels are therefore thought to contribute to changes in microglial activity and/or infiltration. IRF8 is known to regulate the expression of IFN-β, IL-12, iNOS, and related genes [35, 36]. In this study, high IRF8 levels were observed in the retina of rd10 mice. In vitro, we found that LPS insult induced a marked elevation of IRF8 in BV2 cells as well as an increase in the number of iNOS^+^ microglia, suggesting a key role for IRF8 in microglial activation. Strikingly, FA reduced IRF8 and p-STAT1 levels, suggesting that FA suppresses microglial activation partly by regulating IRF8 expression. Additional research on the mechanistic basis of microglial activation and the regulatory role of FA in this process has the potential to offer further insight into the process and prevention of neuroinflammation and to further elucidate the therapeutic utility of FA as an agent for the treatment of neurodegenerative diseases.

## Conclusion

In conclusion, our findings herein suggest that treatment with FA is sufficient to suppress microglial activation in a murine model of RP and thereby markedly attenuates associated neurodegeneration and disease progression. At the mechanistic level, the effect of FA seems to be at least partially linked to its ability to suppress STAT1 activation and IRF8 expression. These results thus highlight the immunomodulatory and anti-inflammatory properties of FA, suggesting that this compound or its derivatives may have value for the treatment of retinal degeneration.

## Supplementary Information

**Additional file: Supplementary figure 1**. FA suppressed iNOS expression in LPS stimulated BV2 cells. **Supplementary table 1**. FA suppressed NO expression in activated BV2 cells. **Supplementary figure 2**. original blot of our research.

## Data Availability

The datasets used and analysed in the current study are available from the corresponding author upon reasonable request.

## Abbreviations

RP: Retinitis pigmentosa
FA: Ferulic acid
IRF8: Interferon regulatory factor 8
TCM: Traditional Chinese medicine
