# Supplementary material for: Ferulic acid attenuates microglia-mediated neuroinflammation in retinal degeneration
Source: BMC Ophthalmol. 2021 Jan 6;21:13. doi: 10.1186/s12886-020-01765-7 (PMC7789661; doi:10.1186/s12886-020-01765-7)
Supplement: Supplementary file 1 — Additional file: Supplementary figure 1. FA suppressed iNOS expression in LPS stimulated BV2 cells. Supplementary table 1. FA suppressed NO expression in activated BV2 cells. Supplementary figure 2. original blot of our research. [file 12886_2020_1765_MOESM1_ESM.docx]

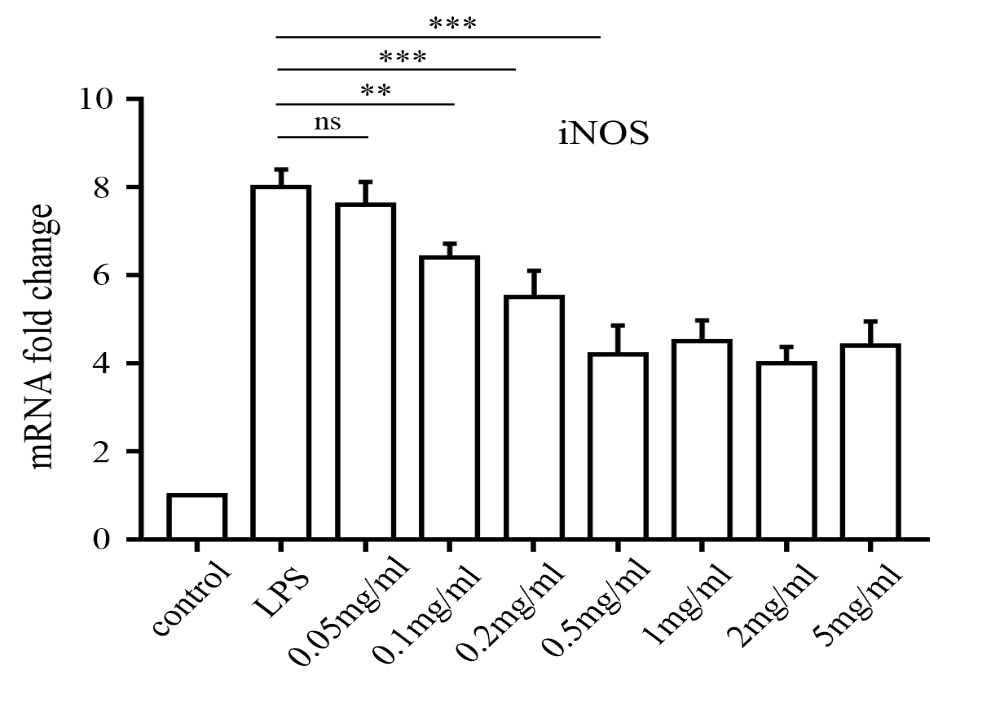


**Supplementary figure 1. FA suppressed iNOS expression in LPS stimulated BV2 cells.** To choose optimal dose for treating microglia cells in vitro. We set FA concentration gradient: 0.05 mg/mL, 0.1 mg/mL, 0.2 mg/mL, 0.5mg/ml, 1mg/ml, 2mg/ml and 5mg/ml. INOS mRNA in BV2 cells was detected to confirm the optimal dose and used for further investigation. As shown in the figure below, 0.5 mg/mL is minimal dose while achieving the best therapeutic effect. (**p<0.01, ***p<0.001, n=6).

**Supplementary table 1. FA suppressed NO expression in activated BV2 cells**

|  | LPS | 0.05FA | 0.1FA | 0.2FA | 0.5FA | 1.0FA | 2.0FA | 5.0FA |
| --- | --- | --- | --- | --- | --- | --- | --- | --- |
| NO Average value (μg/ml) | 86.43 | 80.35 | 73.47 | 68.56 | 50.64 | 51.56 | 52.23 | 51.87 |
| Inhibition rate  (FA-LPS/LPS) |  | 7.03% | 15.0% | 20.68% | 41.40% | 40.34% | 39.56% | 39.98% |

LPS (50ng/ml) stimulated BV2 for 1h, and then FA of different concentration (0.05 mg/mL, 0.1 mg/mL, 0.2 mg/mL, 0.5mg/ml, 1mg/ml, 2mg/ml and 5mg/ml) treated for 24h. According to Griess method, we detected NO concentration in BV2 supernatant.


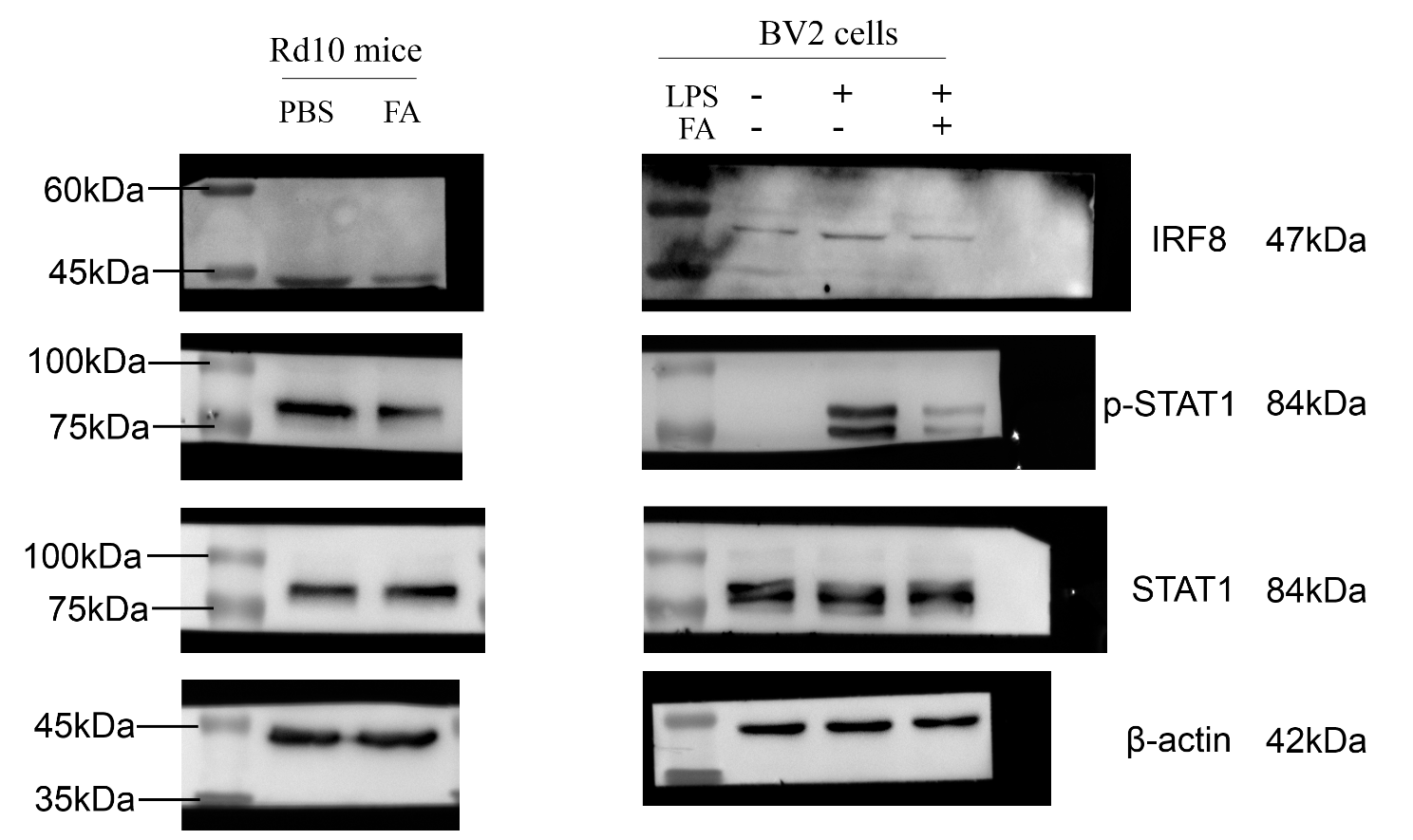


**Supplementary figure 2. original blot of our research.** FA modulated IRF8 activation and phosphorylation of STAT1 in microglia in vivo and vitro.
